# IL-6 as new prognostic factor in patients with advanced cutaneous squamous cell carcinoma treated with cemiplimab

**DOI:** 10.1186/s12967-023-03971-5

**Published:** 2023-02-23

**Authors:** Domenico Mallardo, Ester Simeone, Lucia Festino, Marilena Tuffanelli, Vito Vanella, Claudia Trojaniello, Maria Grazia Vitale, Margaret Ottaviano, Mariaelena Capone, Gabriele Madonna, Francesca Sparano, Eleonora Cioli, Luigi Scarpato, Marco Palla, Rossella Di Trolio, Paolo Meinardi, Corrado Caracò, Gerardo Ferrara, Paolo Muto, Ernesta Cavalcanti, Paolo Antonio Ascierto

**Affiliations:** Istituto Nazionale Tumori – IRCCS – Fondazione “G. Pascale”, Naples, Italy

**Keywords:** Cutaneous squamous cell carcinoma, Cemiplimab, IL-6, Prognostic factor

## Abstract

**Background:**

Prognostic factors for initial response of advanced cutaneous squamous cell carcinoma to cemiplimab treatment are lacking. Il-6 has been found to affect immune cell populations which impact tumor development. The aim was to investigate the prognostic significance of IL-6 serum levels before and during treatment.

**Methods:**

Serum levels of IL-6 were correlated with clinical outcomes in a retrospective study.

**Results:**

Overall, 39 patients were enrolled. High serum levels of IL-6 (> 5.6 pg/ml) were associated with poorer survival (45.1% vs 0 deaths; OS: 16.1 ± 1.5 vs 20.8 ± 0 months, 95% CI 13,046 to 19,184) and shorter PFS (10.3 ± 1.9 vs 18.9 ± 1.5 months; 95% CI 3433 to 10,133) in patients with advanced CSCC treated with cemiplimab. In addition, patients whose IL-6 level increased after treatment with cemiplimab, independently of the basal level, had a poorer response to treatment than patients whose level was reduced or stable after immunotherapy.

**Conclusions:**

Serum levels of IL-6 at baseline and changes after cemiplimab immunotherapy may have a prognostic significance in patients with advanced cutaneous squamous cell carcinoma.

**Supplementary Information:**

The online version contains supplementary material available at 10.1186/s12967-023-03971-5.

## Background

Cutaneous squamous cell carcinoma (CSCC) is the second most common skin cancer in white-skinned populations. The advanced disease develops in less than 5% of patients, but it is difficult to treat and often has a poor prognosis. Immune checkpoint blockade with cemiplimab, a PD-1 inhibitor, has been approved for advanced/metastatic CSCC [1]. However, criteria for indication of cemiplimab in advanced disease are debated, and predictive factors for initial response to cemiplimab treatment are lacking [2].

CSCC frequently develops in chronically inflamed skin and chronic lesions, including burns, wounds, and ulcers [3]. Soluble inflammatory mediators and growth factors, such as Interleukin 6 (IL-6), IL-8 and tumor necrosis factor-α (TNF-α) are known to promote cell proliferation, inhibit apoptosis, activate survival pathways, and recruit leukocytes to the tumor site [4]. IL-6 is a key inflammatory molecule secreted by M2 macrophages after polarization, mediating the progression of pancreatic cancer [5]. The effect of IL-6 on proliferation, metastasis, and tumor immune evasion of colorectal cancer has been demonstrated in recent years [6]. Based on these considerations, we investigated the prognostic significance of serum IL-6 in patients with advanced CSCC undergoing cemiplimab treatment; serum IL-6 levels assessed at baseline and after treatment with cemiplimab were correlated with oncological outcomes in a retrospective study.

## Patients and methods

### Study design

A retrospective study was carried out at Istituto Nazionale Tumori—IRCCS—Fondazione ‘‘G. Pascale,’’ Naples, Italy, upon communication to the local Ethical Committee [protocol n. 32/22 oss]. The study was performed in accordance with the revised version of the Declaration of Helsinki (52nd WMA General Assembly, Edinburgh, Scotland, October 2000).

Adult patients with histologically confirmed unresectable and/or metastatic CSCC, treated with the anti-PD-1 agent cemiplimab, either first- or second-line therapy, were enrolled between July 2020 and February 2022. All patients provided written informed consent.

### Evaluation of outcomes

RECIST 1.1 criteria were used to evaluate the tumor response as complete response (CR), partial response (PR), stable disease (SD), or progressive disease (PD). The following parameters were recorded: serum IL-6 levels; response rate at first assessment; progression-free survival (PFS; defined as the time from administration of the first dose of anti-PD-1 agent to documented radiological progression, death, or lost to follow-up, whichever occurred first); overall survival (OS; defined as the time from administration of the first dose of anti-PD-1 agent to death or lost-to-follow-up, whichever occurred first); disease control rate (DCR; defined as the sum of CR, PR, and SD > 1 year); objective response rate (ORR; defined as the sum of CR and PR); Eastern Cooperative Oncology Group performance status (ECOG PS); American Joint Committee on Cancer (AJCC) distant metastases category; lactate dehydrogenase level.

IL6 was measured by Electrochemiluminescence immunoassays (ECLIA) from Cobas C6000 (Roche) using standard procedures. The serum level of IL-6 was assessed at baseline and 3 months after the start of treatment with cemiplimab, at the time of first response evaluation. A ratio between pre—and post-values was calculated; a ratio > 1 indicates an increase in IL-6 concentrations, while a ratio ≤ 1 indicates decreased or constant levels of IL-6. The cut-off value for IL6 was assessed through the Youden’ s J index which maximizes sensitivity and specificity in a ROC curve. Serum level of IL-6 was classified as high when > 5.6 pg/ml. ROC curves were designed to evaluate the sensitivity/specificity of the cut-off value for PFS (AUC = 0.732) and OS (AUC = 0.591).

### Statistical analysis

Demographic and clinical data were tabulated using descriptive statistics. PFS was calculated as the time from randomization until objective tumor progression or death, whichever occurs first; OS was calculated from as the time from randomization until death by any cause.

The mean and median survival time are reported with their 95% confidence interval (CI). The mean survival time is estimated as the area under the survival curve in the interval 0 to tmax (7). The median survival is the smallest time at which the survival probability drops to 0.5 (50%) or below. If the survival curve does not drop to 0.5 or below then the median time cannot be computed. The median survival time and its 95% CI is calculated according to Brookmeyer & Crowley (8).

Wald test and χ2 log test were used to evaluate the association of variables.

## Results

Overall, 39 patients were enrolled. Demographic and clinical characteristics are reported in Table 1. The median age was 76.7 years (range, 39–96 years), and 31 (79.5%) patients were males. Cemiplimab was used as first-line therapy in 28 (71.8%) patients and as second-line in 11 (28.2%) subjects; nine (81.8%) patients had received radiotherapy prior to immunotherapy, one (9.1%) had received targeted therapy, and one (9.1%) had received immunotherapy other than cemiplimab. The most frequent comorbidities were blood hypertension (64%) and angina/coronary artery disease (31%). Eight patients had low serum IL-6 levels, and 31 had high serum levels. At the first follow-up visit, 15 (38.5%) patients achieved PR, 10 (25.6%) reached SD, and 14 (38.5%) were in progression.

**Table 1:** Demographic and clinical characteristics of enrolled patients (n = 39)

Patient characteristics	n (%)
Median age (years)	76.7 (range 39–96)
Gender: female/male	8 (20.5)/31 (79.5)
CNS metastases at baseline	5 (12.2)
Type of anti-PD-1 agent	
Cemiplimab	39 (100)
Line of treatment	
Frist-line treatment	28 (71.8)
Second-line treatment	11 (28.2)
Type of previous therapy	
Radiotherapy	9 (81.8)
Targeted therapy	1 (9.1)
Immunotherapy	1 (9.1)
Response rate at first assessment	
Partial response	15 (38.5)
Stable disease	10 (25.6)
Progression disease	14 (35.9)
Objective response rate	15 (38.5)
Disease control rate	18 (46.1)
BORR	17 (44%)
*Therapies after PD*	
BSC	16 (41)
chemotherapy	3 (8)
Lost follow up	3 (8)
Comorbidities	
Myocardial infarction	2 (5)
Angina/coronary artery disease	12 (31)
Arrhythmias	9 (23)
Hypertension	25 (64)
Respiratory system	1 (3)
Hepatic	2 (5)
Renal system	4 (10)
Diabetes	8 (21)
Neuromuscular disease	1 (3)
Malignancy: leukemia and myeloma	2 (5)
Malignancy: other cancers	8 (12)
Benign prostatic hyperplasia	4 (10)
Dyslipidemia	9 (23)

*BSC* best supportive care, *BORR* best objective response rate

**Fig. 1:**
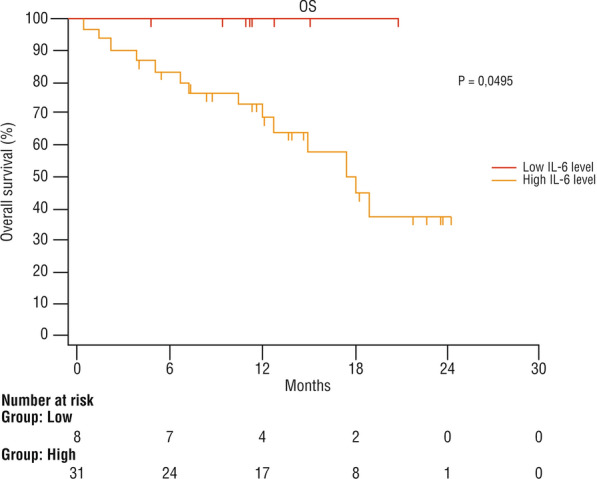
Overall survival after immunotherapy with cemiplimab according to the serum level of IL-6 at baseline

No death was observed in the low serum level group, while 14/31 (45.1%) patients with high IL-6 levels died. The mean OS was 17.4 months in the whole population, 20.8 months in the low IL-6 level group, and 16.1 months in the high IL-6 level group (95% CI 13,046–19,184 months). The Kaplan–Meier curve showed a significantly higher OS in the low-level IL-6 group than in the high-level group (Fig. 1).

**Fig. 2:**
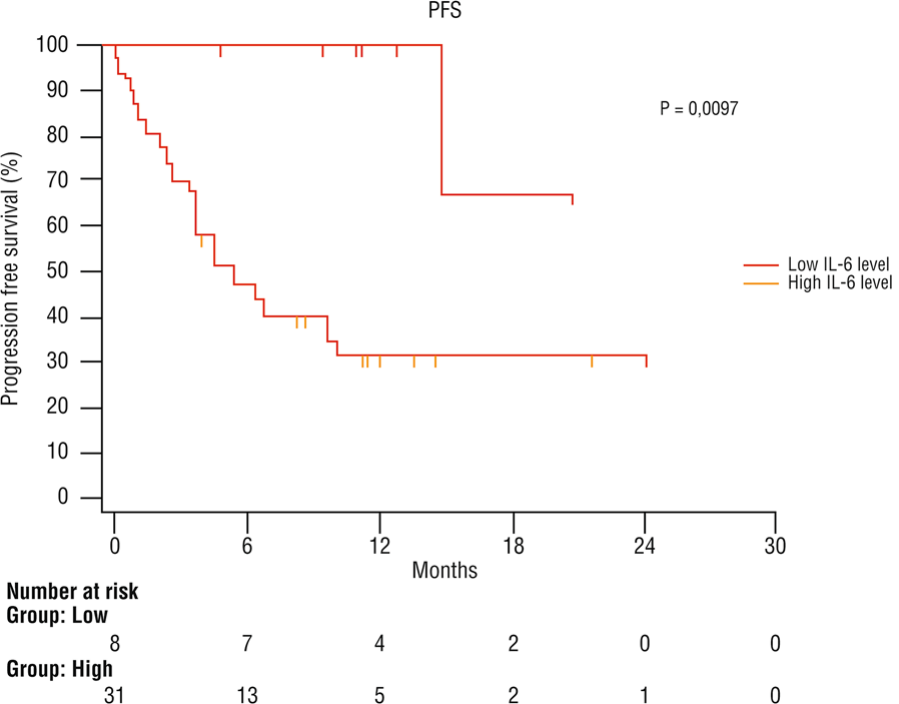
Progression-free survival after immunotherapy with cemiplimab according to the serum level of IL-6 at baseline

The mean PFS was 12.4 months in the overall population, 18.9 months in the low-level IL-6 group, and 10.3 months in the high-level group (95% CI 3433–10,133 months) (Fig. 2).

**Fig. 3:**
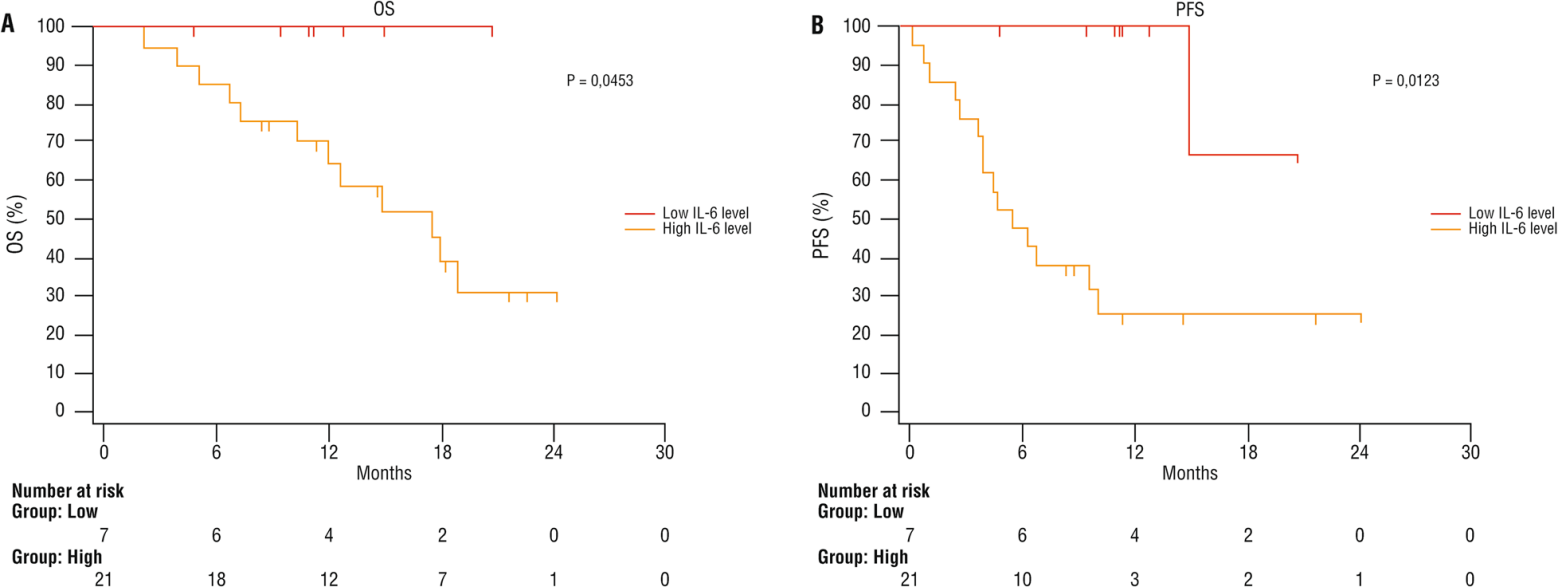
Overall survival (**A**) and PFS (**B**) after immunotherapy with cemiplimab according to the serum level of IL-6 at baseline, in patients naïve to immunotherapy

Similar results were obtained in naïve patients treated in first line with cemiplimab. Median OS was higher in the low-level IL-6 group (20.8 months vs 15.6 months95% CI NA), as was PFS (18.9vs 9.6, 95% CI 0.09317–0.7492, p = 0.012) (Fig. 3A, B).

The multivariate analysis showed that serum IL-6 level was correlated with PFS and was an independent predictive factor for response to cemiplimab (Table 2).

**Table 2:** Multivariate analysis for PFS

Covariate	p-value	HR	95% CI of Exp(b)
Gender	0.489	1.5719	0.4368 to 5.6570
IL6	0.02*	12.957	1.5025 to 111.7340
ORR	0.013*	0.1795	0.0464 to 0.6948
previous treatment	0.295	1.9075	0.5701 to 6.3831

* Correlation is significant at the 0.05 level

**Fig. 4:**
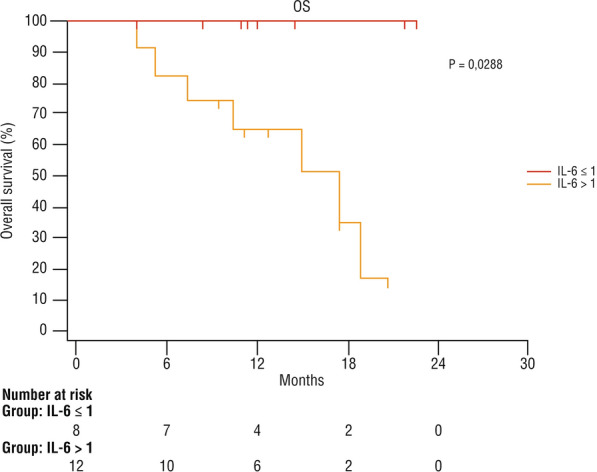
Overall survival according to change of serum IL-6 level after immunotherapy with cemiplimab

**Fig. 5:**
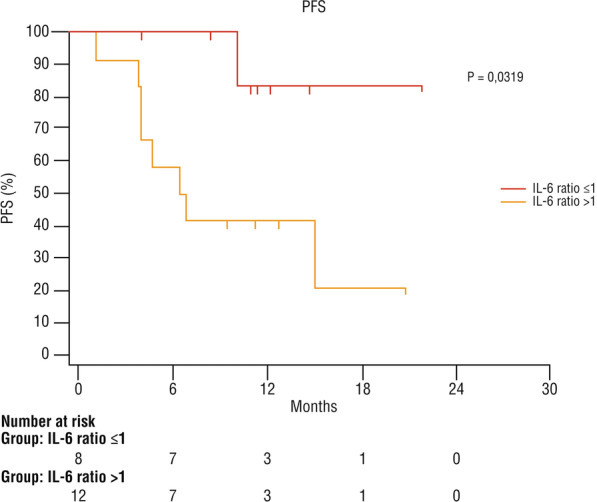
PFS according to change of serum IL-6 level after immunotherapy with cemiplimab

The post/pre cemiplimab ratio of serum levels of IL-6 was calculated in 20 patients (Additional file 1: Figure S1). Eight of them had a ratio ≤ 1 and 12 had a ratio > 1 (Additional file 2: Figure S2). The OS was significantly higher in patients with a ratio ≤ 1 (p = 0.028) (Fig. 4). No death was observed in the group with a ratio ≤ 1, and 7/12 (58%) patients died among those with a ratio > 1. The mean PFS was 19.8 months in patients with a ratio ≤ 1, and 10 months in those with a ratio > 1 (95% CI 3800–15,033) (Fig. 5).

## Discussion

Data from this retrospective study showed that high serum levels of IL-6 were associated with poorer survival and shorter PFS in patients with advanced CSCC treated with cemiplimab. In addition, patients whose IL-6 level increased after treatment with cemiplimab, independently of the basal level, had a poorer response to treatment than patients whose level was reduced or stable after immunotherapy.

Our results seem to agree with Li et al. findings showing that plasma levels of IL-6 and IL-8 in cancer patients with metastases were higher than those without metastases (p < 0.05) in a group of 134 patients with different types of cancer. However, according to our knowledge, we are unaware of studies on a cohort of patients with advanced CSCC [9].

On the other end, we are unaware of previous studies demonstrating a predictive role of serum IL-6 in advanced CSCC treated with immunotherapy. Recently, experimental evidence suggested that IL-6 expression may impact the response to immunotherapy. IL-6 is known to be involved in cell proliferation, immune evasion, and progression of squamous cell cancer [6]. Li et al. found that IL-6 deficient (IL-6^−/−^) mice were less susceptible to developing experimental colorectal cancer than wild-type mice [6]. Additionally, the blockade of IL-6 activated CD8^+^ T-cell accumulation and led to elevated PD-L1 expression in cancers, suggesting possible sensitization to anti-PD-1 therapy.

IL-6 has been proposed as a possible negative prognostic factor in cancer as it has several effects on immune cell populations which impact tumor development; it regulates myeloid-derived suppressor cells (MDSCs) accumulation and activation, resulting in suppression of anti-tumor T cell and natural killer cell function, and stimulation of tumor cell proliferation, survival, invasiveness, and metastasis [10, 11].

The prognostic and predictive role of IL-6 in patients with metastatic melanoma treated with checkpoint inhibitor immunotherapy has been investigated in three large phase II/III randomized trials [12]. In Checkmate-064 (NCT01783938), in both combined cohorts receiving nivolumab and then ipilimumab or the reverse, baseline IL-6 below the median value (13.3 pg/mL) was associated with better OS compared with above the median. On-treatment levels of IL-6 at week 13 were also associated with OS. Checkmate-066 (NCT01721772) and Checkmate-067 (NCT01844505) showed that high baseline levels of IL-6 were associated with poor prognosis in groups receiving nivolumab, ipilimumab, or dacarbazine. In addition, in all three arms of the Checkmate-067 study, IL-6 was a significant independent variate for OS. Overall, the response to checkpoint inhibitor immunotherapy seems to be related to IL-6 baseline and on-treatment levels in several tumor types, possibly with the activation of common mechanisms (6).

This study has some limitations. The number of patients is limited due to the rarity of the condition and blood samples could not be obtained from some patients during the treatment due to management issues. Additionally, basal and on-treatment serum levels of IL-6 were associated with clinical oncologic outcomes of patients with CSCC treated with cemiplimab, our study was not designed to demonstrate a causal role of IL-6. We nevertheless explored the relationship of IL-6 levels with adverse events (AEs). Patients who had AEs during the treatment had a better OS than patients without AEs (p = 0.04; Additional file 3: Figure S3), while there was not a statistically significant difference for PFS (p = 0.06, Additional file 4: Figure S4). It was further observed that patients with cutaneous AEs (rash or itching) were a subgroup with best outcomes. Additionally, low levels of IL-6 at baseline and on-treatment were associated with the occurrence of cutaneous AEs (baseline n = 13, on-treatment n = 10; p < 0.005 [Mann Whitney]) compared to patients who had not AEs (baseline n = 17, o-treatment n = 9). These data suggest that the activation of non-inflammatory immune mechanisms may be correlated with the development of cutaneous AEs, and more importantly with better treatment response. Among patients with low IL-6 level at baseline, 7/8 were naïve to immunotherapy; this latter feature could stand for a higher probability of response to treatment.

Notwithstanding limitations, as data suggest that IL-6 serum level could be a prognostic factor of the response to cemiplimab, this study opens to new investigations. Further studies should include a larger cohort, and conjointly analyze several inflammatory factors such as IL-8, IL-10 and TNF-α.

In conclusion, we found that changes in serum IL-6 levels after cemiplimab immunotherapy have prognostic significance in patients with advanced CSCC and that high levels at baseline correlate with poor response to treatment.

## Supplementary Information

**Additional file 1: Figure 1.** IL-6 level of each patient at baseline. Values over cut-off are represented in red.

**Additional file 2: Figure 2.** Ratio of IL-6 level after/before treatment in each patient. Ratios >1 are represented in red.

**Additional file 3: Figure 3. **OS in patients with or without AEs.

**Additional file 4: Figure 4.** PFS in patients with or without AEs.

## Data Availability

Data and material have been deposited and are publicly available at 10.5281/zenodo.7354525.

## Abbreviations

AJCC: American joint committee on cancer
CR: Complete response
CSCC: Cutaneous squamous cell carcinoma
ECOG PS: Eastern cooperative oncology group performance status
MDSC: Myeloid-derived suppressor cell
OS: Overall survival
PD: Progressive disease
PFS: Progression-free survival
PR: Partial response
SD: Stable disease

## References

[1] Goodman DT (2022). Cemiplimab and cutaneous squamous cell carcinoma: from bench to bedside. JPRAS Open.

[2] Argenziano G, Fargnoli MC, Fantini F, Gattoni M, Gualdi G, Pastore F (2022). Identifying candidates for immunotherapy with cemiplimab to treat advanced cutaneous squamous cell carcinoma: an expert opinion. Ther Adv Med Oncol.

[3] Mueller MM (2006). Inflammation in epithelial skin tumours: old stories and new ideas. Eur J Cancer.

[4] Grivennikov SI, Greten FR, Karin M (2010). Immunity, inflammation, and cancer. Cell.

[5] He Z, Wang J, Zhu C, Xu J, Chen P, Jiang X (2022). Exosome-derived FGD5-AS1 promotes tumor-associated macrophage M2 polarization-mediated pancreatic cancer cell proliferation and metastasis. Cancer Lett.

[6] Li W, Wu Z, Meng W, Zhang C, Cheng M, Chen Y (2022). Blockade of IL-6 inhibits tumor immune evasion and improves anti-PD-1 immunotherapy. Cytokine.

[7] Klein JP, Moeschberger ML (2003). Survival analysis.

[8] Brookmeyer R, Crowley J (1982). A confidence interval for the median survival time. Biometrics.

[9] Li T, Liu M, Dai H, Li X, Liao J, Zheng Z (2022). Value of cytokine expression in early diagnosis and prognosis of tumor metastasis. J Oncol.

[10] Xu M, Zhao Z, Song J, Lan X, Lu S, Chen M, Wang Z, Chen W, Fan X, Wu F, Chen L, Tu J, Ji J (2017). Interactions between interleukin-6 and myeloid-derived suppressor cells drive the chemoresistant phenotype of hepatocellular cancer. Exp Cell Res.

[11] Chen CC, Chen WC, Lu CH, Wang WH, Lin PY, Lee KD, Chen MF (2010). Significance of interleukin-6 signaling in the resistance of pharyngeal cancer to irradiation and the epidermal growth factor receptor inhibitor. Int J Radiat Oncol Biol Phys.

[12] Laino AS, Woods D, Vassallo M, Qian X, Tang H, Wind-Rotolo M (2020). Serum interleukin-6 and C-reactive protein are associated with survival in melanoma patients receiving immune checkpoint inhibition. J Immunother Cancer..

