# Supplementary figures and images for: IL-6 as new prognostic factor in patients with advanced cutaneous squamous cell carcinoma treated with cemiplimab
Source: J Transl Med. 2023 Feb 23;21:140. doi: 10.1186/s12967-023-03971-5 (PMC9948392; doi:10.1186/s12967-023-03971-5)

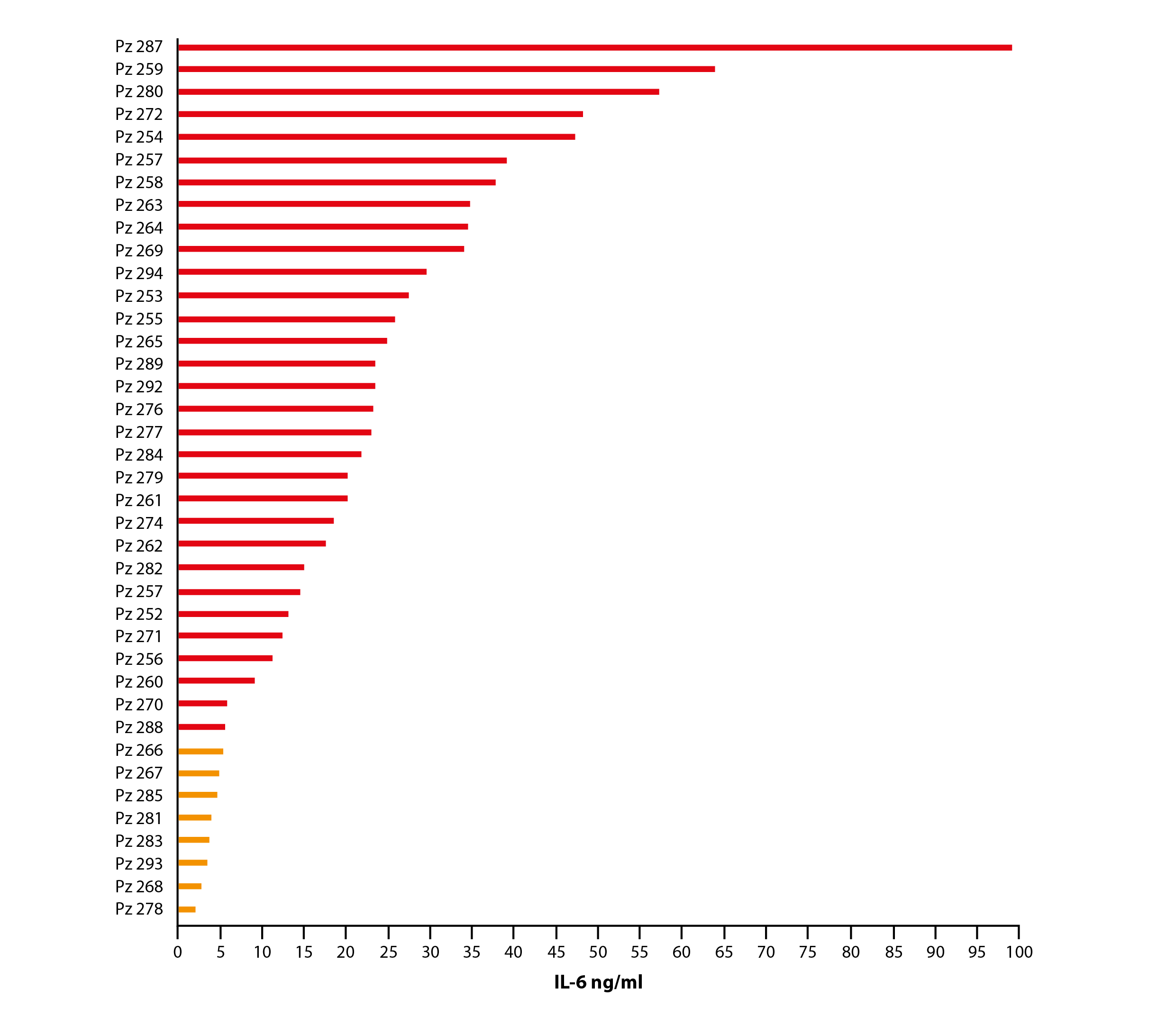

Supplement: Supplementary file 1 — Additional file 1: Figure 1. IL-6 level of each patient at baseline. Values over cut-off are represented in red. [file 12967_2023_3971_MOESM1_ESM.png]

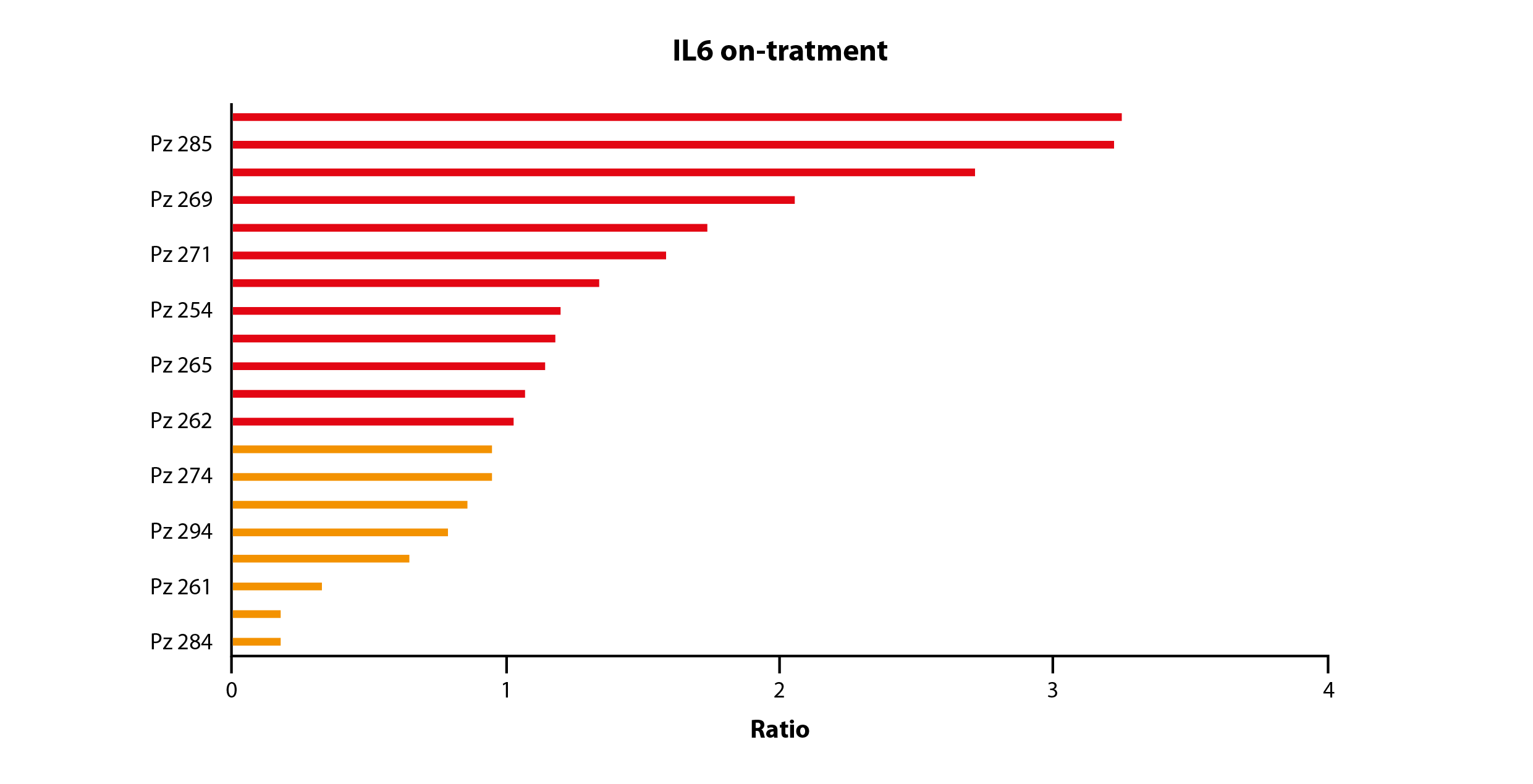

Supplement: Supplementary file 2 — Additional file 2: Figure 2. Ratio of IL-6 level after/before treatment in each patient. Ratios >1 are represented in red. [file 12967_2023_3971_MOESM2_ESM.png]

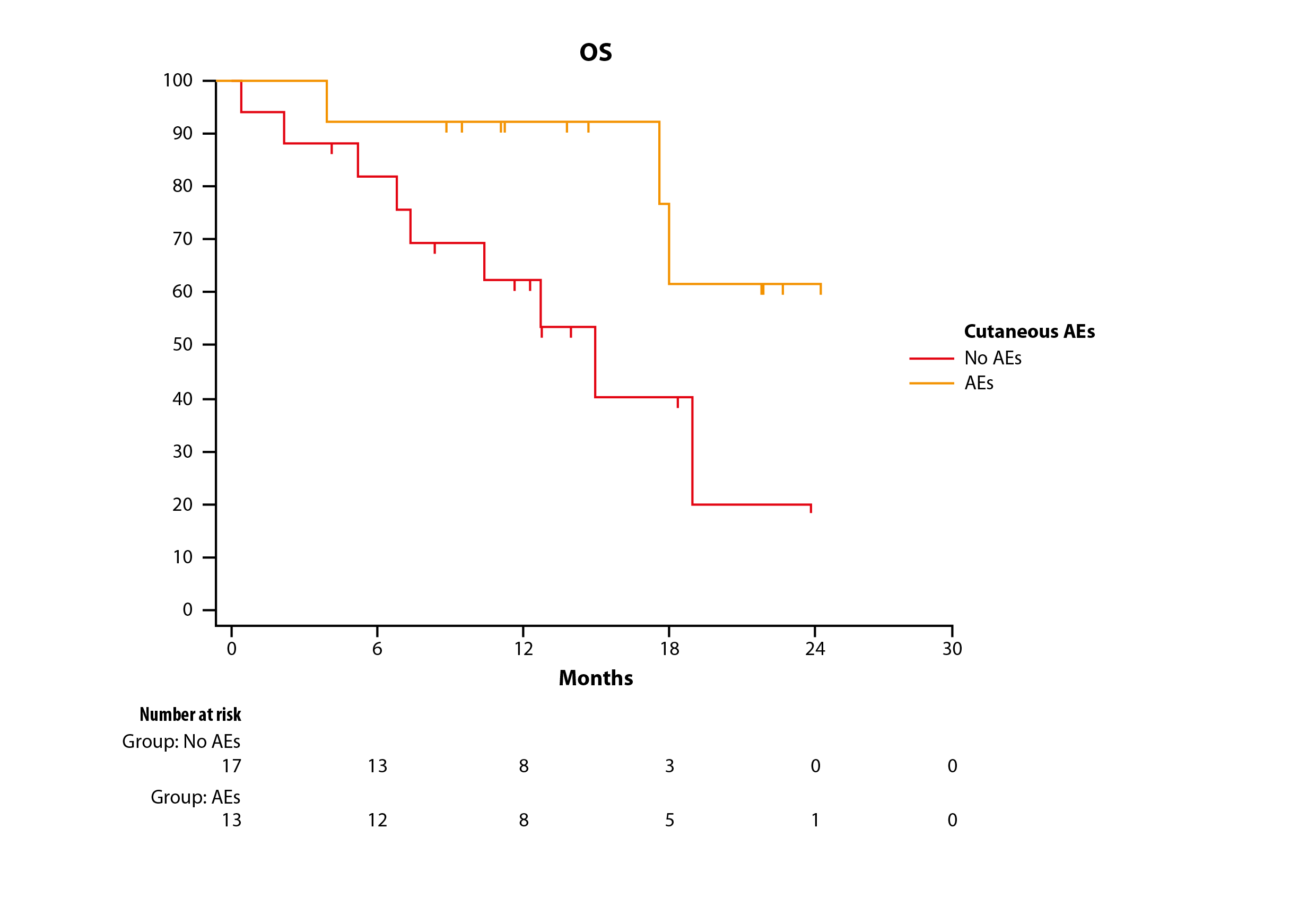

Supplement: Supplementary file 3 — Additional file 3: Figure 3. OS in patients with or without AEs. [file 12967_2023_3971_MOESM3_ESM.png]

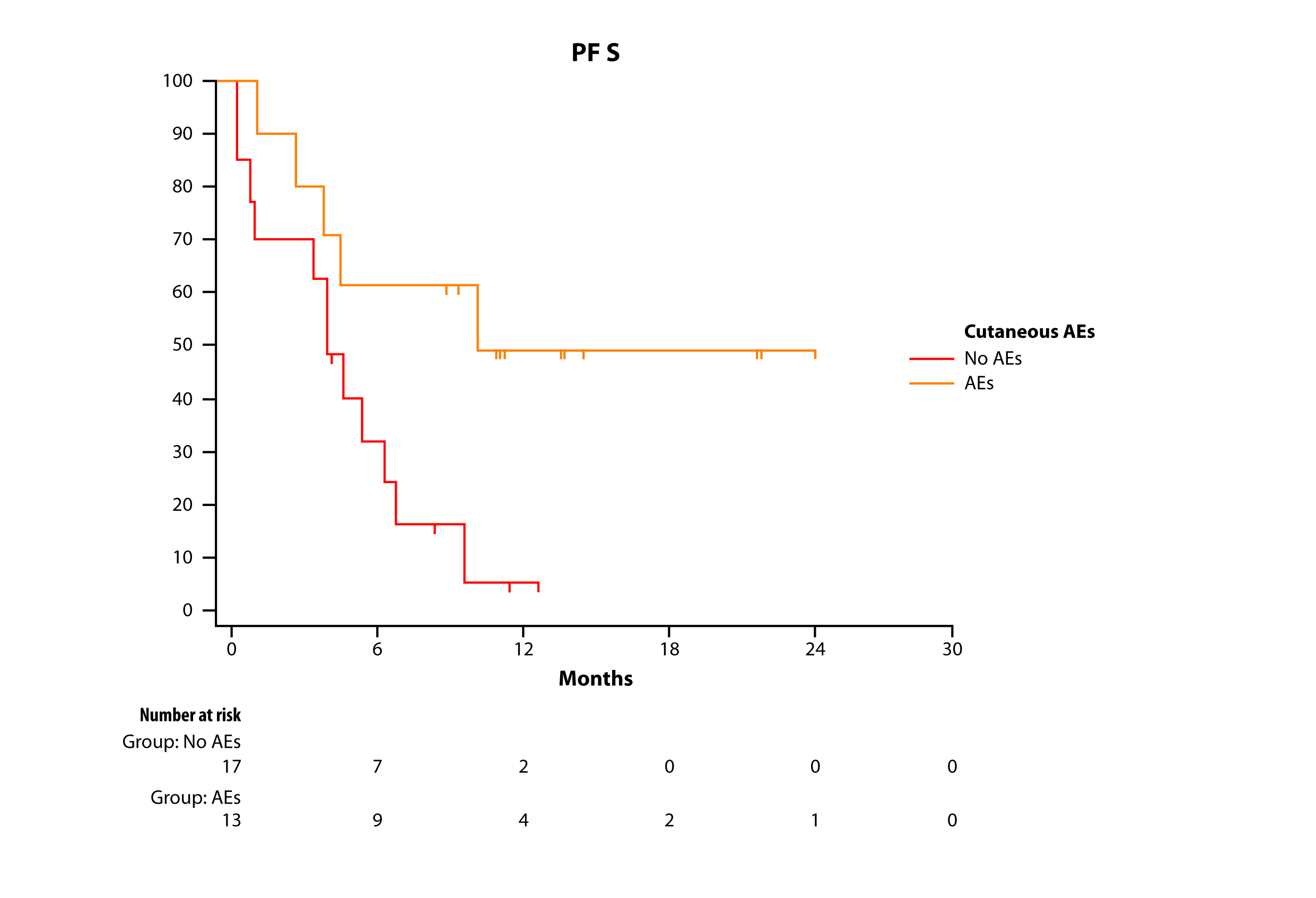

Supplement: Supplementary file 4 — Additional file 4: Figure 4. PFS in patients with or without AEs. [file 12967_2023_3971_MOESM4_ESM.tif]
